# FAME‐04: A Phase 1 trial to assess the safety, acceptability, pharmacokinetics and pharmacodynamics of film and gel formulations of tenofovir

**DOI:** 10.1002/jia2.25156

**Published:** 2018-08-13

**Authors:** Katherine E Bunge, Charlene S Dezzutti, Craig W Hendrix, Mark A Marzinke, Hans M L Spiegel, Bernard J Moncla, Jill L Schwartz, Leslie A Meyn, Nicola Richardson‐Harman, Lisa C Rohan, Sharon L Hillier

**Affiliations:** ^1^ Department of Obstetrics, Gynecology, and Reproductive Sciences University of Pittsburgh Pittsburgh PA USA; ^2^ Magee‐Womens Research Institute Pittsburgh PA USA; ^3^ Division of Clinical Pharmacology Department of Medicine Johns Hopkins University School of Medicine Baltimore MD USA; ^4^ Department of Health and Human Services Kelly Government Solutions Contractor to National Institute of Allergy and Infectious Diseases National Institutes of Health Rockville MD USA; ^5^ CONRAD Eastern Virginia Medical School Arlington VA USA; ^6^ Alpha StatConsult Damascus MD USA; ^7^ Department of Pharmaceutical Sciences University of Pittsburgh Pittsburgh PA USA

**Keywords:** tenofovir, microbicide, vaginal film, vaginal gel, prevention

## Abstract

**Introduction:**

Fast‐dissolving vaginal film formulations release antiretroviral drugs directly into vaginal fluid and may be as efficient at drug delivery yet more acceptable to women than gels. In this Phase 1 vaginal film study, the safety, acceptability, pharmacokinetics and pharmacodynamics of two doses of tenofovir (TFV) film and TFV 1% gel were compared to corresponding placebo formulations.

**Methods:**

Seventy‐eight healthy HIV negative women were randomized to self‐insert daily vaginal film (10 mg TFV, 40 mg TFV or placebo) or 4 mL of vaginal gel (TFV 1% [40 mg] or placebo) for seven days. Grade 2 and higher adverse events (AEs) related to study product were compared across study arms using Fisher's exact test. Plasma TFV concentrations were measured before and 2 hours after last product use. Paired cervical and vaginal tissue biopsies obtained 2 hours after the last dose were measured to determine tenofovir diphosphate (TFV‐DP) concentrations and exposed to HIV in an *ex vivo* challenge assay. Acceptability was assessed through questionnaire.

**Results:**

There was only one grade 2 or higher related AE, the primary endpoint; it occurred in the placebo gel arm. AEs occurred in 90% of participants; the majority (91%) were grade 1. AEs were similar across study arms. TFV concentrations in plasma and TFV‐DP concentrations in cervical and vaginal tissues were comparable between 40 mg TFV film and the TFV gel groups. There was a significant relationship between reduced viral replication and TFV‐DP concentrations in cervical tissues. Film users were less likely to report product leakage than gel users (66% *vs*. 100%, *p* < 0.001).

**Conclusions:**

Films were safe and well tolerated. Furthermore, films delivered TFV to mucosal tissues at concentrations similar to gel and were sufficient to block HIV infection of genital tissue *ex vivo*.

## 1 Introduction

Preexposure prophylaxis (PrEP) with oral Truvada^®^ (emtricitabine 200 mg/tenofovir [TFV] disoproxil fumarate 300 mg) reduces HIV acquisition in women with high adherence to the daily medication 1. One randomized, placebo controlled trial (CAPRISA 004) of vaginal TFV gel demonstrated efficacy 2 with 5.6% HIV incidence in the TFV gel arm compared to 9.1% in the placebo arm (incidence rate ratio = 0.61; *p* = 0.017). Subsequent analyses demonstrated that women with higher TFV concentrations in the cervicovaginal fluid had better protection 3 as did women with a *Lactobacillus*‐dominant vaginal microbiome 4. The MTN‐003 trial (VOICE) assessed the efficacy of daily TFV 1% gel 5. In subsequent analyses utilizing pharmacokinetic (PK) data, the relative risk for HIV infection among those in the TFV gel arm who had detectable concentrations in plasma was 0.53 (*p* = 0.038) 6. In the FACTS 001 trial 7, there was no protective effect seen in the TVF gel coital gel arm; however, a nested case‐cohort analysis of 214 participants showed that detection of TFV in genital fluids was associated with a reduction in HIV acquisition (relative risk = 0.48%; *p* = 0.04) 7.

Understanding that adherence is central to PrEP effectiveness, developing products that are easy to use and support high adherence is important. Efforts have been directed towards developing antiretroviral long acting formulations, such as injectable rilpivirine and cabotegravir, which are being evaluated in clinical trials (clinicaltrials.gov NCT02165202 and NCT03164564 respectively). Vaginal rings containing the antiretroviral drug dapivirine have completed phase three testing 8, 9.

Not all women who need an HIV prevention product would choose a long‐acting product because they may desire HIV protection only during times of sexual activity; therefore, there is still a recognized need for on‐demand microbicide products. Vaginal film formulations of topical microbicides for on‐demand use may provide an attractive alternative to gels by delivering equivalent amounts of drug to the tissues without the leakage associated with the use of gel products. Preclinical studies of maraviroc, tenofovir, dapivirine and emtricitabine containing films support adequate dissolution and drug release from the film matrix 10. In the first human study of a fast‐dissolving antiretroviral (ARV) containing film, dapivirine concentrations in plasma and genital samples were comparable between women randomized to daily film and gel use for a week 11. This study provided proof of concept that ARV films could deliver drug as efficiently as gel thus supporting further investigations, including research into extended release platforms and drug‐containing nanoparticles embedded into film platforms. To date, a 40‐mg TFV film has been evaluated in a single dose in one study and was found to deliver TFV at similar or higher concentrations than the gel formulation 12. The current study is a Phase 1 trial of the same TFV vaginal film. In this multi‐dose study of vaginal TFV, the safety, acceptability, PK and pharmacodynamics (PD) were compared between TFV 1% gel, two doses of TFV film and the corresponding placebos.

## 2 Methods

This was a Phase 1, five arm, single‐site, double blind, randomized placebo‐controlled trial comparing the safety and acceptability of TFV 1% gel and two doses of TFV film to corresponding placebos. The study was conducted in accordance with the ethical standards of the University of Pittsburgh Institutional Review Board and in accordance with the Helsinki Declaration of 1975, as revised in 2000. All participants provided written informed consent prior to initiation of study procedures.

Seventy‐eight healthy, nonpregnant HIV‐uninfected women 18 to 45 years of age were enrolled between January and December 2014. Participants were required to be on an effective method of contraception for 30 days prior to enrolment. Women were excluded if they reported vaginal symptoms or were found to have *Neisseria gonorrhoeae* and *Chlamydia trachomatis*. Women were randomized with equal frequency to one of five arms using a permuted block design with block sizes of 10. The groups included 4 mL universal placebo gel (HEC gel) 13, 4 mL TFV 1% gel, placebo film, TFV (10 mg) film and TFV (40 mg) film. The TFV films were two by two inches in size and contained hydroxypropyl methyl cellulose, hydroxyethylcellulose, sodium carboxymethylcellulose and glycerine; the TFV 1% gel was HEC based.

There were four study visits. Eligibility was assessed at the screening visit during which a baseline 10 mL saline cervicovaginal lavage (CVL) was obtained. Eligible participants presented for the enrolment visit within 56 days of screening. After baseline samples were collected, participants self‐inserted the first dose of study product while in the clinic. Participants were provided with five additional doses of product to use daily at home. Six days later, participants had a third clinic visit at which point they had a pre‐dose plasma sample and vaginal swab collected. The last dose of study product was inserted in the clinic. Two hours later, participants underwent specimen collection, including plasma, CVL, vaginal swab, rectal swab and genital tissue biopsies (two cervical and two vaginal biopsies). Participants returned one month after enrolment for a final safety assessment visit.

The primary objective was to assess safety. The primary endpoint was grade 2 or higher Adverse Events (AEs) deemed related to study product. All participants were included in the safety analysis. Local and systemic safety was assessed by eliciting AEs through history, physical exam and laboratory evaluation. AEs were defined and graded per the Division of AIDS Table for Grading the Severity of Adult and Paediatric AEs, Version 1.0, Updated August 2009 and the Female Genital Grading Table for Use in Microbicide Studies (Addendum 1 to the DAIDS Table for Grading Adult and Paediatric Adverse Events, Version 1.0, November 2007). The number of AEs by body system and relationship to study product was tabulated; individual participants contributed only once to the calculation of event rates. The proportion of participants experiencing grade 2 and higher AEs deemed related to study product was compared across treatment arms using Fisher's exact tests.

Secondary objectives included changes in cervico‐vaginal ecology and TFV concentrations in plasma, cervicovaginal fluid (CVF), rectal fluid and CVL, as well as both TFV and tenofovir diphosphate (TFV‐DP) concentrations in genital tissues. For the pharmacokinetic (PK) analyses and the exploratory objectives only, women who were assessed as evaluable and who had evidence of study product placement at the time of the biopsies were included. Participants were evaluable if they reported not missing two or more home doses and using the dose on the day prior to the biopsy visit. One set of tissue biopsies for quantitative drug analysis was washed, weighed, snap frozen and stored at −80°C for drug quantification. All pharmacokinetic measurements were acquired via validated liquid chromatographic‐tandem mass spectrometric (LC‐MS/MS) as previously described 12. The LLOQs for TFV in plasma and CVL were 0.31 ng/mL and 5 ng/mL respectively 14. For samples that were below the LLOQ of TFV and TFV‐DP, the concentration was assumed to be half the LLOQ for plasma, CVL, cervicovaginal and rectal fluid samples and half the LLOQ adjusted for average weight of the cervical and vaginal tissue samples. To evaluate the impact of product on vaginal microbiota, Nugent scores, quantitative vaginal cultures, and quantitative polymerase chain reaction (PCR) tests for selected microbes were compared between groups using Fisher's exact and Kruskal‐Wallis tests.

The exploratory objectives were to (1) compare the protective effect of TFV gel and TFV film to placebo products against HIV in an *ex vivo* biopsy challenge assay and (2) assess formulation effects on mucosal innate anti‐HIV‐1 activity. A second set of biopsies was used fresh for the *ex vivo* HIV challenge assay. As previously described 15 the tissue biopsies were exposed to HIV‐1_BaL_ and viral replication was monitored using HIV‐1 p24 ELISA (Alliance, Perkin Elmer). A log‐log, linear least‐squared model was used to test for the significance of the slope estimate, difference from zero, where a statistically significant negative slope indicated drug‐mediated virus suppression. The LLOQ values were imputed for TFV‐DP of 3.173 fmol/mL. Log_10_ TFV‐DP and log_10_ cumulative p24 antigen (from *ex vivo* HIV challenge) were fit to a 4‐parameter non‐linear logistic PK‐PD model (Eq. 1).

(1) $$y=a+\frac{b-a}{1+10^{LogIC50-x*\mathrm{Hillslope}}}$$

where *a* and *b* are the lower and upper asymptotes of the curve respectively. Using the parameter estimates from the PK‐PD equation, the 90% effective TFV‐DP tissue concentration (EC_90_) for the *ex vivo* challenge test was calculated. For CVL PD activity, the assessment was performed in an *in vitro* TZM‐bI assay with the CVL diluted to a final titre of 1:5. The correlation between PD activity and TFV concentration in the CVL was assessed using linear regression. To assess the impact of product formulation on innate antiviral activity, anti‐HIV activity after placebo product use (2 hours after last dose and one month after enrolment) was compared to baseline activity by the Wilcoxon signed‐rank test.

Product acceptability was collected via self‐administered questionnaires after one week of product use and compared using Fisher's exact tests.

The sample size was based on the exact binomial probability of observing at least 2 adverse events. For a given arm, if the true rate of a given toxicity endpoint was 5%, 14 women per arm would provide 85% power to exclude toxicity endpoint rates greater than 30%. Fourteen women per arm assured that a 95% confidence interval for the difference between the placebo and TFV toxicity rates had an upper limit no more than 16% when the observed toxicity rates for placebo and active gel were both 5%. Non‐evaluable participants were replaced. Additional participants were screened and enrolled.

## 3 Results

**Figure 1:**
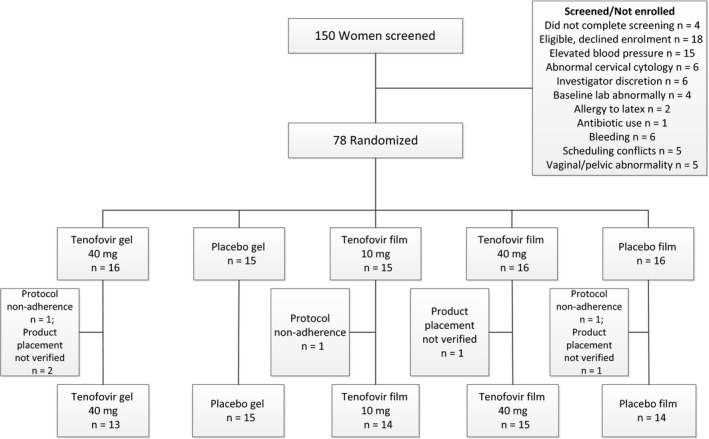
The disposition of participants and how each participant contributed to different analyses.

One hundred and fifty women were screened, and 78 women enrolled. The primary reasons for screen failure are outlined in the CONSORT diagram (Figure 1).

Fifteen women were randomized to 10 mg TFV film, 16 to 40 mg TFV film, 16 to placebo film, 16 to TFV 1% gel and 15 to placebo gel. Three participants were non‐evaluable and replaced. Four additional participants were included in the safety evaluation but were not included in the secondary or exploratory outcomes (2 gel and 2 film users) because product placement could not be verified. Specifically, at the time of the biopsy, two women randomized to film were noted to have film distal to the vaginal introitus and two women randomized to gel had no evidence of gel in the vagina. Of the 234 scheduled clinic visits, 233 (99.6%) were completed. No differences with respect to demographic characteristics and sexual behaviour were noted between study arms (Table 1).

**Table 1:** Demographic and behavioural characteristics of the study population

Characteristic	Gel Placebo (n = 15)	Gel Tenofovir 40 mg (n = 16)	Film Placebo (n = 16)	Film Tenofovir 10 mg (n = 15)	Film Tenofovir 40 mg (n = 16)	Total (N = 78)	*p*‐value
Race, n (%)							0.89^a^
White, non‐Hispanic	12 (80%)	12 (75%)	11 (69%)	12 (80%)	10 (62%)	57 (73%)	
Black, non‐Hispanic	2 (13%)	3 (19%)	4 (25%)	2 (13%)	5 (31%)	16 (21%)	
Black, Hispanic	0	0	0	1 (7%)	0	1 (1%)	
Asian	1 (7%)	0	1 (6%)	0	1 (6%)	3 (4%)	
Bi‐racial	0	1 (6%)	0	0	0	1 (1%)	
Age, years (SD)	27.1 (7.5)	29.5 (5.7)	27.4 (6.1)	25.6 (5.4)	28.9 (5.0)	27.7 (6.0)	0.40^b^
Body mass index, kg/m^2^ (SD)	25.4 (3.5)	25.8 (4.4)	28.5 (7.5)	29.7 (9.0)	28.9 (9.0)	27.7 (7.1)	0.34^b^
Education							0.49^a^
High school graduate or less	0	1 (6%)	3 (19%)	2 (13%)	2 (12%)	8 (10%)	
Some college or college graduate	11 (73%)	8 (50%)	5 (31%)	8 (53%)	9 (56%)	41 (53%)	
At least some post‐graduate education	4 (27%)	7 (44%)	8 (50%)	5 (33%)	5 (31%)	29 (33%)	
Unmarried	9 (60%)	8 (50%)	10 (62%)	12 (80%)	7 (44%)	46 (59%)	0.30^a^
Current smoker	2 (13%)	4 (25%)	2 (12%)	3 (20%)	2 (12%)	13 (17%)	0.82^a^
At least one prior pregnancy	4 (27%)	6 (38%)	7 (44%)	5 (33%)	8 (50%)	30 (38%)	0.74^a^
Sexually active past 30 days, with male partner	9 (60%)	10 (62%)	8 (50%)	9 (60%)	7 (44%)	43 (55%)	0.81^a^

a *p*‐value from Fisher's exact test.

b *p*‐value from one‐way analysis of variance.

The primary endpoint was grade 2 or higher related AEs. There was only one such AE for grade 2 vaginal pain which was reported in the placebo gel arm. This participant reported vaginal pain and was noted to have had two retained gel applicator caps in her vagina. Ninety percent of participants reported at least 1 AE during study follow‐up (Table 2). Most AEs were grade 1 (91%). The most commonly reported AE was product leakage, which was more common in the gel group (*p* = 0.007). There was a similar distribution of mild and moderate events across the five groups (Table 2) except for vaginal discharge which was more frequently reported in the 40 mg TFV film group (*p* = 0.008). Six women in the 40 mg TFV group reported abnormal vaginal discharge; all were grade 1. Four of these events involved a brownish discharge, three of which started during the week of product use and the fourth after the biopsy visit. Neither abnormal vaginal discharge nor mucosal irritation or ulceration was noted on pelvic exam with one exception; one participant had vulvar erythema and discharge and was ultimately diagnosed with vulvovaginal candidiasis.

**Table 2:** Incidence of Adverse Events (AEs) by study arm

	Total (N = 78)	Gel Placebo (n = 15)	Gel Tenofovir, 40 mg(n = 16)	Film placebo (n = 16)	Film Tenofovir, 10 mg (n = 15)	Film Tenofovir, 40 mg (n = 16)	*p*‐value^a^
Participants with at least one AE	70 (89.7%)	14 (93.3%)	15 (93.8%)	14 (87.5%)	13 (86.7%)	14 (87.5%)	0.98
*Gastrointestinal complaint*	9 (11.5%)	3 (20.0%)	0	1 (6.3%)	3 (20.0%)	2 (12.5%)	0.30
Abdominal discomfort	3 (3.8%)	1 (6.7%)	0	0	1 (6.7%)	1 (6.3%)	0.64
Nausea/Emesis	5 (6.4%)	1 (6.7%)	0	1 (6.3%)	1 (6.7%)	2 (12.5%)	0.80
Bowel dysfunction	6 (7.7%)	1 (6.7%)	1 (6.3%)	2 (12.5%)	1 (6.7%)	1 (6.3%)	>0.99
*Genitourinary complaint*	62 (79.5%)	13 (86.7%)	15 (93.8%)	12 (75.0%)	9 (60.0%)	13 (81.3%)	0.21
Product leakage	52 (65.8%)	13 (86.7%)	14 (82.4%)	11 (68.8%)	6 (40.0%)	8 (50.0%)	0.025
Pelvic pain	7 (9.0%)	1 (6.7%)	1 (6.3%)	1 (6.3%)	1 (6.7%)	3 (18.8%)	0.81
Genital itching	6 (7.7%)	1 (6.7%)	2 (12.5%)	0	1 (6.7%)	2 (12.5%)	0.79
Genital irritation	7 (9.0%)	1 (6.7%)	2 (12.5%)	2 (12.5%)	1 (6.7%)	1 (6.3%)	>0.99
Vaginal discharge	11 (14.1%)	2 (13.3%)	1 (6.3%)	1 (6.3%)	1 (6.7%)	6 (37.5%)	0.008
Vaginal odour	4 (5.1%)	1 (6.7%)	0	2 (12.5%)	0	1 (6.3%)	0.73
Vaginal infection	4 (5.1%)	1 (6.7%)	1 (6.3%)	0	1 (6.7%)	1 (6.3%)	0.91
Bleeding abnormality	8 (10.3%)	1 (6.7%)	2 (12.5%)	1 (6.3%)	3 (20.0%)	1 (6.3%)	0.73
AE Severity (any body system)							
Any grade 1	70 (89.7%)	14 (93.3%)	15 (93.8%)	14 (87.5%)	13 (86.7%)	14 (87.5%)	0.98
Any grade 2	11 (14.1%)	1 (6.7%)	2 (12.5%)	1 (6.3%)	3 (20.0%)	4 (25.0%)	0.54
Any grade 3	1 (1.3%)	0	0	0	0	1 (6.3%)	–
AE relatedness to study product (any body system)							
Any related	58 (74.4%)	13 (86.7%)	15 (93.8%)	11 (68.8%)	8 (53.3%)	11 (68.8%)	0.07
Any not related	40 (51.3%)	7 (46.7%)	8 (50.0%)	8 (50.0%)	9 (60.0%)	8 (50.0%)	0.96

Each participant contributes only one observation per category.

a *p*‐value from Fisher's exact test.

Participants randomized to use the 40 mg TFV film and TFV 1% gel had trough plasma TFV concentrations after 6 doses [median, IQR] of 1.84 ng/mL (0.46, 2.81) and 0.86 ng/mL (0.40, 1.72) respectively (Table 3). When comparing the two 40 mg formulations, both TFV and TFV‐DP concentrations were similar in all matrices – plasma, CVF, rectal fluid, cervical and vaginal tissue ‐ apart from higher TFV CVF with film compared to gel (Table 3). TFV with 40 mg film was 2.0‐times higher pre‐dose (*p* = 0.052) and 2.9‐times higher 2 hours post‐dose (*p* < 0.001) when compared to 40 mg gel.

**Table 3:** Tenofovir and tenofovir diphosphate concentrations in biologic matrices following use of 1% tenofovir gel containing 40 mg of tenofovir and vaginal films containing 40 or 10 mg of tenofovir

	1% TFV Gel (n = 13)	40 mg Film (n = 15)	*p*‐value^a^	10 mg Film (n = 14)	*p*‐value^b^	40:10 mg Film Ratio
Tenofovir (ng/mL)						
Plasma TFV trough after 6 doses	0.86 (0.40, 1.72)	1.84 (0.46, 2.81)^a^	0.17	0.40 (0.16, 0.61)	0.007	4.6
Plasma TFV 2 hours after 7th dose	2.34 (1.38,4.75)	2.74 (0.85, 5.31)^a^	0.96	0.98 (0.39, 1.52)	0.007	2.8
Cervicovaginal lavage 2 hours after 7th dose	193 x 10^3^ (138 x 10^3^, 608 x 10^3^)	181 x 10^3^ (114 x 10^3^, 320 x 10^3^)	0.39	72.5 × 10^3^ (56.1 × 10^3^, 100 × 10^3^)	0.001	2.5
Tenofovir (ng/mg)						
Cervicovaginal fluid TFV trough after 6 doses	531.97 (311.24, 622.03)	1043.86 (446.85, 2170.75)	0.052	337.58 (89.38, 1463.61)	0.16	3.1
Cervicovaginal fluid TFV 2 hours after 7th dose	2.85 x 10^3^ (2.07 x 10^3^, 3.57 x 10^3^)	8.34 x 10^3^ (4.00 x 10^3^, 11.54 x 10^3^)	<0.001	1.63 x 10^3^ (0.60 x 10^3^, .52 x 10^3^)	<0.001	5.1
Rectal fluid TFV 2 hours after 7th dose	33.67 (3.19, 832.22)	33.99 (15.04, 228.33)	0.75	14.90 (9.49, 67.58)	0.17	2.3
TFV‐DP concentration (fmol/mg)						
Cervical tissue 2 hours post dose 7	222.08 (70.96, 555.64)	936.91 (55.84, 1456.45)	0.27	35.20 (23.16, 113.46)	0.001	26.6
Vaginal tissue 2 hours post dose 7	295.90 (149.89, 917.14)	241.05 (113.32, 545.89)	0.44	50.47 (22.89, 221.15)	0.046	4.8

Data presented as median (interquartile range). Drug concentrations were assessed at two time points. Participants presented to the clinic after using the study product for six doses. Specimens were collected for trough PK at the beginning of the visit. Participants inserted the seventh dose in the clinic and two hours later, additional samples were collected (7th dose).

a *p*‐value from Mann‐Whitney *U* test for difference between 40 mg TFV film and 1% TFV gel.

b *p*‐value from Mann‐Whitney *U* test for difference between 40 mg TFV film and 10 mg TFV film.

Between the two film formulations, dose‐proportionality was approximately equivalent for the 40 mg:10 mg dose ratio, ranging from 2.3 to 5.1 across matrices. Only cervical tissue, which demonstrated very high variability within each formulation, fell outside this range with a ratio of 26.6 for the film.

Because microbiota associated with bacterial vaginosis (BV) have previously been reported to decrease tenofovir in vaginal fluid 4, the baseline prevalence of BV and the frequency and levels of lactobacilli and BV‐associated bacteria was evaluated and found to be similar across the treatment arms (Table 4). The impact of product use on the microbiota was assessed after women received 7 doses. There were no statistically significant changes in the microbiota based on cultivation and PCR based methods following product use with the exception of a decrease in the prevalence (*p* = 0.031) and quantity (*p* = 0.011) of *Prevotella bivia* in the 10 mg film group (data not shown).

**Table 4:** Vaginal microbiota of women at baseline

Microbiota	Gel Placebo (n = 15)		Gel Tenofovir, 40 mg (n = 13)		Film Placebo (n = 14)		Film Tenofovir, 10 mg (n = 14)		Film Tenofovir, 40 mg (n = 15)		*p*‐value^b^
	n (%) positive	Quantity^a^ median (range)	n (%) positive	Quantity^a^ median (range)	n (%) positive	Quantity^a^ median (range)	n (%) positive	Quantity^a^ median (range)	n (%) positive	Quantity^a^ median (range)	
Bacterial vaginosis^c^	1 (6.7%)		1 (7.7%)		4 (28.6%)		5 (35.7%)		5 (33.3%)		0.17
*Lactobacillus. crispatus* d	12 (80.0%)	7.5 (6.9 to 7.9)	10 (76.9%)	7.5 (3.0 to 8.0)	8 (57.1%)	7.6 (6.8 to 7.9)	8 (57.1%)	7.6 (7.1 to .8)	10 (66.7%)	7.6 (6.4 to 7.8)	0.59
*Lactobacillus iners* d	8 (53.3%)	7.0 (3.8 to 8.6)	6 (46.2%)	6.3 (3.6 to 7.9)	9 (64.3%)	6.9 (5.5 to 8.3)	10 (71.4%)	7.7 (2.9 to 8.5)	11 (73.3%)	6.6 (3.4 to 8.7)	0.55
*Lactobacillus jensenii* d	7 (46.7%)	8.1 (5.6 to 8.6)	7 (53.8%)	6.7 (5.1 to 8.4)	9 (64.3%)	6.7 (4.0 to 7.9)	7 (50.0%)	6.6 (3.4 to 8.0)	6 (40.0%)	6.3 (4.6 to 7.4)	0.79
*Lactobacillus gasseri* d	5 (33.3%)	5.6 (4.2 to 6.6)	7 (53.8%)	5.2 (3.0 to 7.6)	4 (28.6%)	5.1 (3.5 to 6.9)	6 (42.9%)	5.6 (3.1 to 6.7)	3 (20.0%)	5.7 (5.4 to 5.9)	0.40
*Lactobacillus vaginalis* d	7 (46.7%)	7.1 (5.7 to 7.9)	7 (53.8%)	5.9 (5.0 to 7.3)	8 (57.1%)	5.8 (5.3 to 7.5)	7 (50.0%)	6.0 (5.1 to 6.5)	5 (33.3%)	6.0 (5.3 to 7.6)	0.77
*Gardnerella vaginalis* d	5 (33.3%)	4.6 (4.0 to 8.6)	7 (53.8%)	5.1 (3.8 to 7.9)	7 (50.0%)	6.4 (4.3 to 8.2)	11 (78.6%)	6.9 (4.2 to 8.0)	9 (60.0%)	6.9 (3.5 to 8.1)	0.18
*Atopobium vaginae* d	2 (13.3%)	6.5 (5.2 to 7.8)	2 (15.4%)	6.2 (4.5 to 7.9)	4 (28.6%)	7.7 (6.6 to 9.3)	7 (50.0%)	7.0 (3.9 to 9.2)	6 (40.0%)	8.2 (7.1 to 8.8)	0.16
*Megasphaera* type I^d^	1 (6.7%)	8.0	1 (7.7%)	7.8	4 (28.6%)	7.6 (6.1 to 8.0)	5 (35.7%)	7.5 (6.7 to 8.3)	4 (26.7%)	7.6 (6.9 to 7.9)	0.22
*Prevotella bivia* d	5 (33.3%)	2.6 (2.5 to 4.2)	3 (23.1%)	3.7 (2.9 to 5.3)	3 (21.4%)	2.2 (2.0 to 6.5)	9 (64.3%)	2.8 (2.0 to 7.0)	4 (26.7%)	2.9 (2.2 to 5.9)	0.13
*Prevotella amnii* d	0		0		4 (28.6%)	8.0 (6.3 to 8.5)	4 (28.6%)	6.4 (4.1 to 8.2)	3 (20.0%)	7.8 (7.6 to 8.5)	0.03
*Preotella timonensis* d	5 (33.3%)	4.2 (2.2 to 7.3)	6 (46.2%)	4.2 (3.1 to 7.3)	7 (50.0%)	4.4 (3.0 to 7.0)	11 (78.6%)	4.2 (2.9 to 8.2)	6 (40.0%)	5.5 (3.7 to 8.3)	0.14
Group B *Streptococcus* e	3 (20.0%)	3.3 (3.1 to 3.5)	1 (7.7%)	2.8	0		1 (7.1%)	4.1	3 (20.0%)	3.1 (2.5 to 3.3)	0.34
*Enterococcus* e	3 (20.0%)	2.5 (2.1 to 3.3)	3 (23.1%)	2.6 (2.5 to 3.6)	2 (14.3%)	2.1 (2.1 to 2.1)	1 (7.1%)	3.5	2 (13.3%)	3.5 (2.3 to 4.8)	0.82
*Staphylococcus aureus* e	0		1 (7.7%)	3.3	0		2 (14.3%)	3.4 (3.3 to 3.5)	1 (6.7%)	3.5	0.42
*Escherichia coli* e	1 (6.7%)	2.1	2 (15.4%)	2.1 (2.1 to 2.1)	1 (7.1%)	2.5	3 (21.4%)	3.9 (2.8 to 4.3)	0		0.31
*Candida albicans* e	3 (26.7%)	4.5 (2.6 to 4.9)	2 (15.4%)	4.5 (4.1 to 5.0)	3 (21.4%)	3.1 (2.5 to 3.1)	4 (28.6%)	4.1 (1.1 to 4.9)	3 (20.0%)	4.1 (2.1 to 4.3)	0.96
Other yeast^e^	0		1 (7.7%)	3.9	0		1 (7.1%)	3.1	0		0.34

a Median quantity (range) among positive results; there were no differences in the quantity of microbiota detected by qPCR or culture methods at enrolment (*p* > 0.2 from Kruskal‐Wallis test for all organisms).

b *p*‐value from Fisher's exact test for detection of organism.

c As defined by Nugent score ≥7.

d Organisms detected by qPCR.

e Cultivated organisms.

**Figure 2:**
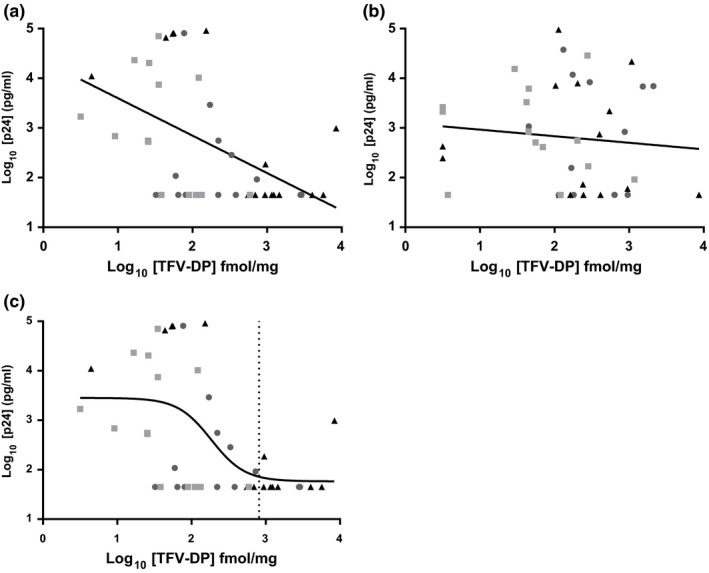
Tenofovir‐diphosphate (TFV‐DP) pharmacokinetic and pharmacodynamic activity in female genital tract tissue. Paired biopsy tissues were exposed to HIV‐1_BaL_ *ex vivo*; there was a significant relationship between HIV p24 antigen production and TFV‐DP concentrations for cervical **(a)** (*p *<* *0.01), but not vaginal **(b)** (*p* = 0.52) tissue. The non‐linear 4‐parameter model (black line) defined the EC_90_ of TFV‐DP in cervical tissue for all treatment groups **(c)** (vertical dotted line). Triangle, TFV 40 mg film; square, TFV 10 mg film; circle, TFV gel

TFV‐DP PK and PD activity in female genital tract tissue was assessed (Figure 2). There was a significant linear relationship (*p *<* *0.01; *r* ^*2 *^= 0.26; slope = −0.75 (±0.20)) between viral replication and TFV‐DP concentrations for cervical (Figure 2a) but not vaginal (*p* = 0.52) tissue (Figure 2b). This linear relationship between the cervical tissue TFV‐DP and viral replication was modelled with a non‐linear, 4‐parameter curve and the 90% effective concentration (EC_90_) of TFV‐DP in cervical tissue was estimated to be 813 fmol/mg (2.91 log_10_, 95% CI was non‐calculable) (Figure 2c).

Because products inserted into the vagina could impact mucosal innate anti‐HIV activity, changes were evaluated over time among placebo users (Table 5). Increased mucosal innate anti‐HIV activity was noted for film users 2 hours after placebo film use compared to baseline; this increase returned to baseline by one month. For women randomized to gel, mucosal innate anti‐HIV‐1 activity significantly decreased from baseline to 2 hours after product use. Despite rebounding partially at one month, mucosal innate anti‐HIV‐1 activity did not return to pre‐product use levels among gel users.

**Table 5:** Innate anti‐HIV‐1 activity in the CVL of women two hours after using placebo film or gel. Data are presented as median (interquartile range)

Product	Mucosal Innate anti‐HIV‐1 activity (% control)		
	Screening	2 hours after 7th dose	1 month visit
Film (n = 14)	70.2 (43.4, 87.4)	85.8 (66.4, 90.8)	69.1 (50.0, 80.8)
*p*‐value^a^		0.013	0.78
Gel (n = 15)	77.7 (41.9, 89.6)	34.8 (12.9, 64.3)	59.8 (−8.3, 77.3)
*p*‐value^a^		0.003	0.036

a *p*‐value from Wilcoxon signed‐rank test comparing innate anti‐HIV‐1 activity assessed at Visits 3 and 4 to innate anti‐HIV‐1 activity assessed prior to product use at Visit 1.

Overall, study participants reported that both film and gel formulations were acceptable (Table 6). While 50% of the women reported some difficulty with film use, only two of 47 participants randomized to film use had evidence of poor product placement on exam. By comparison, two of 31 women randomized to gel had evidence of poor product placement. The major issue participants reported with film placement was film adhering to the finger. Despite nearly half of the film users reporting some difficulty with product usage, 72% of film users and 77% of gel users reported that they would likely use the product should it be found effective against HIV.

**Table 6:** Acceptability

Characteristic	Total (N = 78) (%)	Gel Placebo (n = 15) (%)	Gel Tenofovir, 40 mg (n = 16) (%)	Film Placebo (n = 16) (%)	Film Tenofovir, 10 mg (n = 15) (%)	Film Tenofovir, 40 mg (n = 16) (%)	*p*‐value^a^
How difficult was the product to insert?							0.002
Difficult	25 (32)	1 (7%)	1 (6%)	7 (44%)	8 (53%)	8 (50%)	
Not difficult	53 (68)	14 (93%)	15 (94%)	9 (56%)	7 (47%)	8 (50%)	
How did the study product feel once inserted?							0.25
Uncomfortable	22 (28)	6 (40)	7 (44)	2 (12)	4 (27)	3 (19)	
Not uncomfortable at all	56 (72)	9 (60)	9 (56)	14 (88)	11 (73)	13 (81)	
Did you experience product leakage?							<0.001
No leakage	16 (21)	0	0	3 (19)	8 (53)	5 (31)	
Some leakage	62 (79)	15 (100)	16 (100)	13 (81)	7 (47)	11 (69)	
How likely would you be to use this product if it were found to protect users from getting HIV?							0.14
Unlikely	20 (26)	1 (7)	6 (38)	6 (38)	2 (13)	5 (31)	
Likely	58 (74)	14 (93)	10 (62)	10 (62)	13 (87)	11 (69)	

Gel users reported more product leakage (100%) compared to the film group (66%).

a *p*‐value from Fisher's exact test.

## 4 Discussion

In this evaluation of a vaginal film containing TFV, daily use of two film doses or a vaginal gel yielded comparable safety and tolerability results. The rate of AEs was similar between TFV and placebo arms and similar to rates of AEs published for other vaginal microbicide trials of TFV 5. Vaginal discharge was more frequently reported in the 40 mg TFV group, but objectively abnormal discharge was confirmed by exam in only one woman. Importantly, genital, plasma and tissue concentrations were comparable between the 40 mg TFV film and the 1% TFV gel suggesting equally efficient drug delivery systems. The PK results from this study mirror results from two PK trials of TFV vaginal gel 16, 17. Robinson et al. compared the pharmacokinetics and pharmacodynamics of a single dose of TFV 40 mg film to TFV 1% gel 12. Concentrations of TFV in all matrices were below those observed in the present study which could be due to accumulation of drug with multiple dosing in the present study. In a post‐hoc analysis of CAPRISA 004, tenofovir concentration of ≥100 ng/mL in CVF was associated with 65% (CI: 6%; 87%) protection against HIV, while a ≥ 1000 ng/mL concentration correlated with 76% (CI: 8%; 92%) protection against HIV infection. In this study tenofovir trough concentrations measured in CVF approximately 24 hours after the 6th dose were higher than >100 ng/mL for all formulations; furthermore, the drug concentrations measured two hours after the final product use exceeded 1000 ng/mL for all formulations. However, unlike our daily dosing regimen, CAPRISA 004 used on demand dosing whereby the timing of the dose prior to CVF sampling was not known making comparisons challenging.

Both products achieved TFV‐DP concentrations in cervicovaginal tissue far in excess of concentrations achieved with daily oral dosing 16, itself associated with high levels of HIV protection in women 1. The relative contribution to HIV protection conferred by drug concentrations in local tissue compared to systemic concentrations, however, remains uncertain.

In our study, the TFV‐DP concentrations in cervical tissue biopsies decreased HIV‐1 replication (EC_90_ of 813 fmol/mg; EC_50_ = 204, 61 to 682 95% CI). This was similar to the estimated concentrations of TFV‐DP reported by Nicol et al. who reported an EC_50_ of 715 fmol/mg of TFV‐DP after a single dose of oral Truvada 18. The lack of anti‐HIV response in vaginal tissue could be due to different distribution or ratio of immune cells (HIV‐1 target cells) to other cell types such as epithelial cells, which could have limited the drug available to lymphocytes. Overall, our data show TFV‐DP concentrations achieved through films or gels could suppress HIV‐1 infection *ex vivo*. However, more work is needed to define the distribution of drug, dNTP pools as well as drug transporters and metabolizing enzymes within the female genital tract, which would ultimately affect the potency of TFV‐DP for HIV‐1 suppression 19, 20.

In this study, the mucosal innate anti‐HIV‐1 activity was evaluated longitudinally among placebo users, and women using gel lost significantly more innate activity than film users. The clinical impact of this decrease in innate activity is uncertain. Taken together, however, the ability of the TFV to deliver enough drug to cervical tissue to provide protection against HIV in an *ex vivo* model and the observation that film formulation does not impact innate anti‐HIV activity suggest that the TFV film is a promising topical microbicide option.

As contraceptive research has demonstrated, providing formulation and dosing options for women is critical to increase uptake 21, 22. The film dosage form could provide a lower cost on‐demand microbicide product for women that may be more acceptable than gels and, consequently, may improve adherence.

Acceptability evaluations of vaginal gels have consistently demonstrated that one major drawback to gel use is perceived messiness and leakage. Our study substantiated previous research that found vaginal film products to have less leakage than gel products. Several secondary analyses of the vaginal gel trials have shown that partner disclosure of gel use was associated with significantly higher adherence rates 23, 24. Because films are thin and dry, they have a relatively small volume and weight when compared to aqueous‐based gels. This has two distinct advantages with respect to covert use: the packaging for films is much smaller and the product is associated with less leakage than gels with use.

The acceptability results from our study are encouraging but limited. Nearly half of the women randomized to the film arm reported some difficulty with product placement, and at the time of biopsy, the clinician identified two participants (4.2%) who had not inserted the film completely inside of the vagina. Of note, two participants randomized to gel product also had evidence of poor product placement at the time of biopsy, that is, no evidence of gel in the vagina. While this may represent non‐adherence, a different participant had retained applicator caps in her vagina, suggesting that she had inserted the gel applicator without removing the applicator cap. Therefore, the absence of gel product in the vagina could also represent misuse. The strengths of our study included a randomized study design and excellent participant retention and study conduct. This enabled us to meet the primary and secondary objectives of assessing safety and evaluating drug delivery of film formulations with confidence. A limitation of this study was the short duration of film use, which limited the opportunity for women to increase their familiarity with the dosage form. Other limitations to the trial include the small sample size and short exposure time inherent in Phase 1 trials and the limited generalizability of the study results given that women recruited into the trial understood that they would be undergoing several pelvic exams and using a vaginal product.

## 5 Conclusion

Our study confirmed that tenofovir film is safe and delivers drug to the genital tissues with similar drug concentrations as gel but with less leakage. Films have the potential to be an on‐demand product for women to use prior to vaginal sex. The next step in the advancement of vaginal film technology, developing films with different release patterns that may confer protection for days, rather than hours, should enhance user compliance thereby providing the prospect of better protection against HIV‐1 infection.

## Competing interests

Dr. Hillier reported receiving consulting fees from Merck and Lupin. During the period of the analysis and manuscript preparation, Dr. Hendrix consulted for ViiV/GSK. No other authors report conflicts of interest.

## Authors’ contributions

KB, CD, CH, MM and BM were involved in data collection. KB, CD, CH, MM, BM, LM, NRH, LR and SLH were involved in data analysis KB, CD, CH, MM, BM, LM, NRH, LR, SLH, HS and JS were involved in data interpretation and presentation of results.
